# Gap Junction Intercellular Communication Positively Regulates Cisplatin Toxicity by Inducing DNA Damage through Bystander Signaling

**DOI:** 10.3390/cancers10100368

**Published:** 2018-10-02

**Authors:** Sanjeevani Arora, Joshua R. Heyza, Elaine C. Chalfin, Randall J. Ruch, Steve M. Patrick

**Affiliations:** 1 Department of Cancer Biology, University of Toledo Health Science Campus, Toledo, OH 43614, USA; sanjeevani.arora@fccc.edu (S.A.); echalfin38@gmail.com (E.C.C.); randall.ruch@utoledo.edu (R.J.R.); 2 Department of Oncology, Karmanos Cancer Institute and Wayne State University, Detroit, MI 48201, USA; jrheyza@med.wayne.edu

**Keywords:** connexin 43, gap junction, gap junction intercellular communication, cisplatin, bystander effect, ERCC1-XPF, chemoresistance

## Abstract

The radiation-induced bystander effect (RIBE) can increase cellular toxicity in a gap junction dependent manner in unirradiated bystander cells. Recent reports have suggested that cisplatin toxicity can also be mediated by functional gap junction intercellular communication (GJIC). In this study using lung and ovarian cancer cell lines, we showed that cisplatin cytotoxicity is mediated by cellular density. This effect is ablated when *GJA1* or Connexin 43 (Cx43) is targeted, a gap junction gene and protein, respectively, leading to cisplatin resistance but only at high or gap junction forming density. We also observed that the cisplatin-mediated bystander effect was elicited as DNA Double Strand Breaks (DSBs) with positive H2AX Ser139 phosphorylation (γH2AX) formation, an indicator of DNA DSBs. These DSBs are not observed when gap junction formation is prevented. We next showed that cisplatin is not the “death” signal traversing the gap junctions by utilizing the cisplatin-GG intrastrand adduct specific antibody. Finally, we also showed that cells deficient in the structure-specific DNA endonuclease *ERCC1*-*ERCC4* (ERCC1-XPF), an important mediator of cisplatin resistance, further sensitized when treated with cisplatin in the presence of gap junction forming density. Taken together, these results demonstrate the positive effect of GJIC on increasing cisplatin cytotoxicity.

## 1. Introduction

Cisplatin chemotherapy is effective and currently active against tumor types from various ontogenies, including osteosarcomas, testicular, lung, ovarian, cervical and head and neck cancers [1,2]. Since its discovery by Rosenberg as a potential chemotherapeutic agent nearly 50 years ago, many groups have worked on assessing cisplatin’s mechanism of action. Resistance or variable responses can limit its efficacy, thus underscoring the importance of understanding the mechanisms of cisplatin resistance. Cisplatin reacts with DNA to produce platinum-DNA adducts, including intra- and inter-strand crosslinks (ICLs), and it is well accepted that these lesions mediate cisplatin’s cytotoxic effect [3]. It is well known that resistance to cisplatin and its analogues can arise through multiple mechanisms broadly divided into: (1) Mechanisms that reduced the formation of platinum-DNA adducts such as decreased drug uptake, increased drug efflux, detoxification, or increased/altered DNA repair; and (2) Increased cell survival in the presence of DNA damage due to alterations in the signaling pathways that contribute to apoptosis [4].

Work by Jensen and Glazer has shown that cisplatin treatment could lead to cytotoxicity in neighboring untreated bystander cells through gap junctions (GJs) [5]. Additionally more recent studies have focused on the role of Cx43 in mediating the bystander effect after cisplatin treatment. First, it has been described by several other groups that reduced Cx43 expression is a common event in cell lines that are generated to be resistant to cisplatin, however the actual signal inducing cell death in bystander cell remains unknown [6,7]. Second, evidence showing reduced Cx43 expression appears to be associated with increased resistance to cisplatin has generated interest in the possibility of treating with a GJIC enhancer to enhance platinum efficacy with some promising preclinical results [8]. In addition, this observation is not unique to platinum treatment, but has been observed in various cancer types using multiple anti-neoplastic agents, including gemcitabine, adriamycin, and paclitaxel [9,10,11]. There is a large body of evidence exhibiting enhanced radiation toxicity due to functional gap junctional intercellular communication (GJIC) [12]. It has been shown that this radiation toxicity is due to toxic or “death” signals traversing the gap junction channels. However, we still do not have a clear understanding of these “death” molecules or signals. Some evidence has been provided that these “death” signals likely induce DNA damage in bystander cells and may be related to reactive oxygen and nitrogen species crossing gap junctions [13,14]. However, it is unclear in the context of cisplatin whether cisplatin itself can traverse GJs to induce DNA damage in bystander cells. Interestingly, in experiments blocking GJIC or gap junctions, pharmacologically or by transcript downregulation respectively, it was shown that this “bystander effect” (BE) is dependent on the level of GJIC and that even low levels of GJIC exhibited this BE [14,15].

Gap junctions are a direct connection from cell to cell to transfer molecules such as ions, cyclic AMP, cyclic GMP, phosphoinositides, nucleotides, amino acids, or glutathione. This communication maintains tissue and organ homeostasis and helps in diverse processes like growth, development and differentiation. GJ channels are made of connexin (Cx) proteins with six Cx monomers forming a hemichannel which docks onto a hemichannel from a neighboring cell to complete the gap junction channel [16]. Interestingly, several studies showed that loss of Cx expression or GJIC can be a marker of tumorigenesis. However, other studies also suggested that certain cancers retain GJIC or upregulated GJIC especially when in transition to a metastatic phenotype [17,18].

Jensen and Glazer reported that cells proficient in the non-homologous end joining mechanisms (NHEJ) of DNA repair exhibited sensitivity to cisplatin only when treated at GJ-forming high density [5]. Kalvelyte et al. have shown that functional GJs enhanced cisplatin-mediated apoptosis [18]. In another study by the Glazer group, it was shown that activated src phosphorylated Cx43 which decreased GJIC and increased survival in the presence of cisplatin [19]. In our study, utilizing lung and ovarian cancer cell lines, we tested the impact of gap junction modulation on cisplatin cytotoxicity. Our results suggest a model where cisplatin cytotoxicity is enhanced in the presence of functional GJ interactions. Our data also suggest that GJIC functionality could be useful as a novel strategy to impact, positively, cisplatin chemotherapy and other agents that target DNA repair. 

## 2. Results

### 2.1. Cisplatin Treatment at High Density Sensitizes Cancer Cell Lines

**Figure 1:**
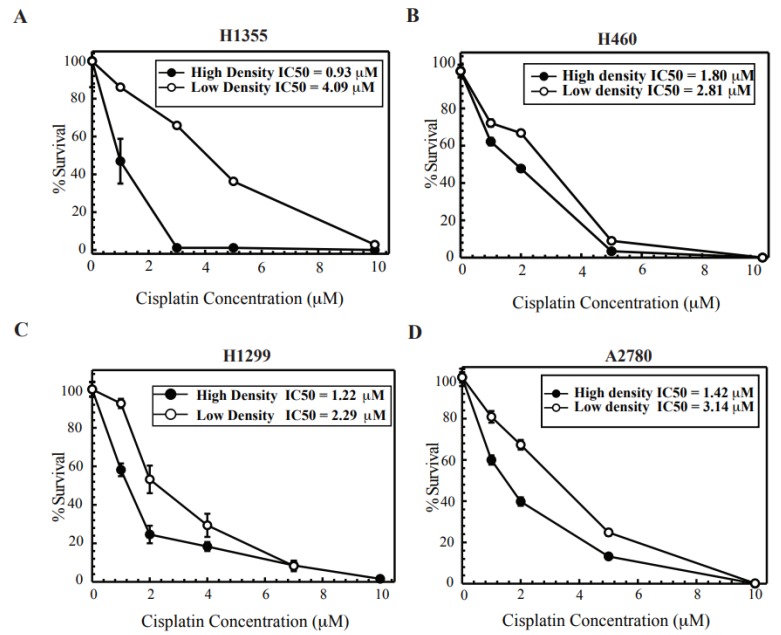
Clonogenic survival after cisplatin treatment at low and high density of cells. (**A**) H1355, (**B**) H460, (**C**) H1299, (**D**) A2780. Clonogenic survival was performed at high-density and low-density cisplatin treatment as described in the methods section. Calculated IC_50_ values are represented in each figure for each cell line. Values are represented as mean ± SEM from three independent experiments.

Lung and ovarian cancer cell lines were tested for density dependent cisplatin cytotoxicity. It has been shown that a high-density confluent monolayer of cells promotes the formation of GJs while at low colony forming density cells are dispersed with no contact to promote GJ formation. This procedure has been used extensively to study the role of GJ-mediated effects [5]. Figure 1, shows the survival of cells exposed to cisplatin for 2 h at low- and high-density conditions (see Section 4 for a description and Supplemental Figure S1 for a schematic representing how these assays were performed). Clonogenic survival is reduced in both conditions, however when cells are treated at high density, the cell survival is reduced below the low-density cells in a concentration-dependent manner. Thus, increased cisplatin cytotoxicity is observed in conditions where there is an opportunity to form GJ channels that could allow GJIC. Figure 1, H1355 (1A) and A2780 (1D) cells showed the maximum fold change in toxicity (~4 fold and ~2.5 fold respectively) with a minimal change in H1299 (1C) and H460 (1B) cells (~1.8 fold and ~1.5 fold change respectively). Next, the trypsinization process post cisplatin treatment could potentially affect survival at high density; hence to test this, we treated cells with cisplatin at colony forming density and re-plated them for colony formation post treatment. Supplemental Figure S2A,B, shows that there is no significant difference in IC_50_ values when cells are trypsinized and re-plated post treatment.

### 2.2. Connexin Expression in Lung and Ovarian Cancer Cells

**Figure 2:**
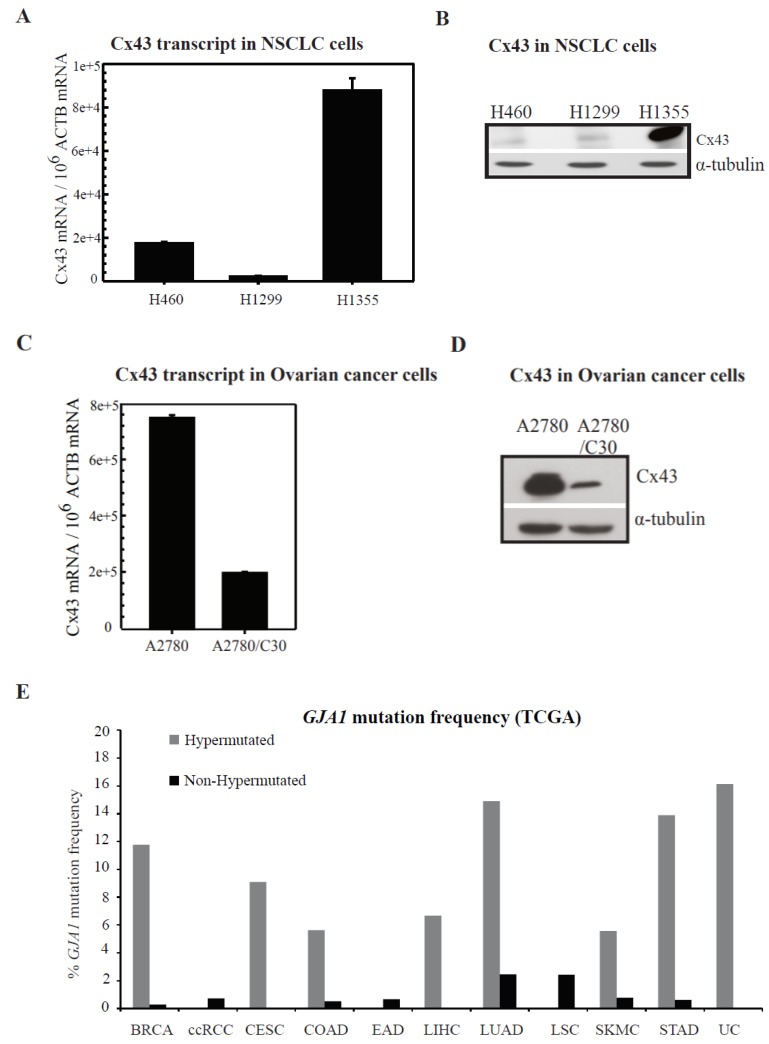

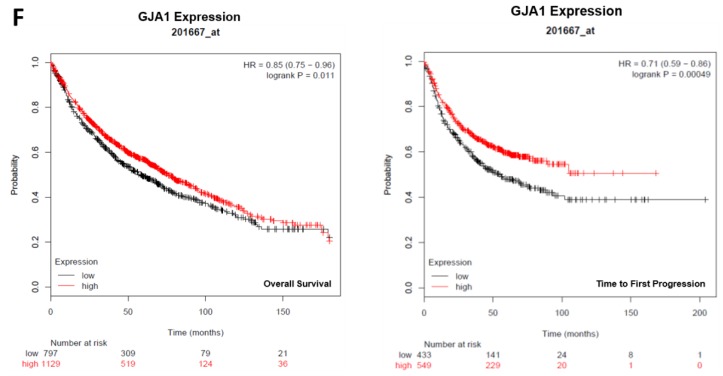
Cx43 in cancer. (**A**–**D**) Cx43 expression in NSCLC and ovarian cancer cells: RNA (**A**,**C**) and protein (**B**,**D**). (**A**,**C**) Total RNA was extracted from cells and analyzed using StaRT-PCR, as described in Section 4. Each PCR was run in triplicate. The transcript levels are represented as Cx43 mRNA/10^6^ ACTB mRNA. The values are represented as mean ± SEM from triplicate PCRs. (**B**,**D**) Whole cell lysate from the cells were probed with antibody for Cx43 with α-tubulin as a loading control. Each PCR was run in triplicate. The transcript levels are represented as Cx43 mRNA/10^6^ ACTB mRNA. The values are represented as mean ± SEM from triplicate PCRs. (**E**) Graph indicates the frequency of *GJA1* somatic mutations in different cancers extracted from cancer studies in the TCGA (The Cancer Genome Atlas) (data retrieval date November 23rd 2016). Cancer abbreviations are BRCA, breast invasive carcinoma; ccRCC, clear cell Renal Cell Carcinoma; CESC, cervical squamous cell carcinoma; COAD, colorectal adenocarcinoma; LIHC, liver hepatocellular carcinoma; LUAD, Lung Adenocarcinoma; LSC, Lung Squamous Carcinoma; SKMC, cutaneous melanoma; STAD, stomach adenocarcinoma; UC, uterine carcinoma. The graph has been divided to indicate mutation frequencies in hypermutated and non-hypermutated cancer. (**F**) Survival plots indicating probability of overall survival and time to first progression in lung cancers based upon GJA1 expression in human tumors obtained from kmplotter.org.

Connexin 43 (Cx43) has been widely studied and shown to be dysregulated in cancer [20,21]. We studied the expression of Cx43 in non-small cell lung cancer cells (NSCLC)—H1355, H1299 and H460. Figure 2A (RNA) and Figure 2B (protein) depicts that H1355 cells highly express Cx43 while H1299 and H460s have a reduced expression at least when compared to H1355. Figure 2C (RNA) and Figure 2D (protein) depicts Cx43 expression in ovarian cancer cell lines. Parental cisplatin sensitive and their resistant counterpart ovarian cancer line A2780 and A2780/C30 (Figure 2C,D) were tested. Cx43 expression is significantly increased in parental cisplatin sensitive cells while interestingly reduced in resistant cells. Somatic mutations identified by The Cancer Genome Atlas (TCGA) and other studies as affecting *GJA1* (Cx43) in cancer are shown in Figure 2E, and indicate specific association of *GJA1* mutations with hypermutated lung adenocarcinomas, where it is detected in ~15% of cases, as well as a strong bias towards mutation in hypermutated stomach, uterine, breast, cervical, colorectal and liver cancers. Additionally, Figure 2F shows how GJA1 expression in lung tumors may influence overall survival as well as time to first progression. These data show that in general, patients with low GJA1 expression have generally worse survival outcomes than those patients whose tumors have high GJA1 expression, particularly in lung cancers. This further supports the idea that GJIC may play an important physiological role in mediating survival in cancers in response to therapy. The Lucifer yellow dye transfer is a commonly employed method to detect the presence of functional GJs and has been extensively used [22]. We performed Lucifer yellow dye-transfer analysis and show that all the cell lines tested were able to communicate the GJ permeant dye, Lucifer yellow. For H1299 and H1355 cells, we also observed that dye transfer is not affected by cisplatin treatment (results summarized in Figure S3D). These data suggest that in these cell lines cisplatin treatment does not affect GJ activity.

### 2.3. Cx43 Knockdown Cells Leads to Cisplatin Resistance at High-Density Treatment

**Figure 3:**
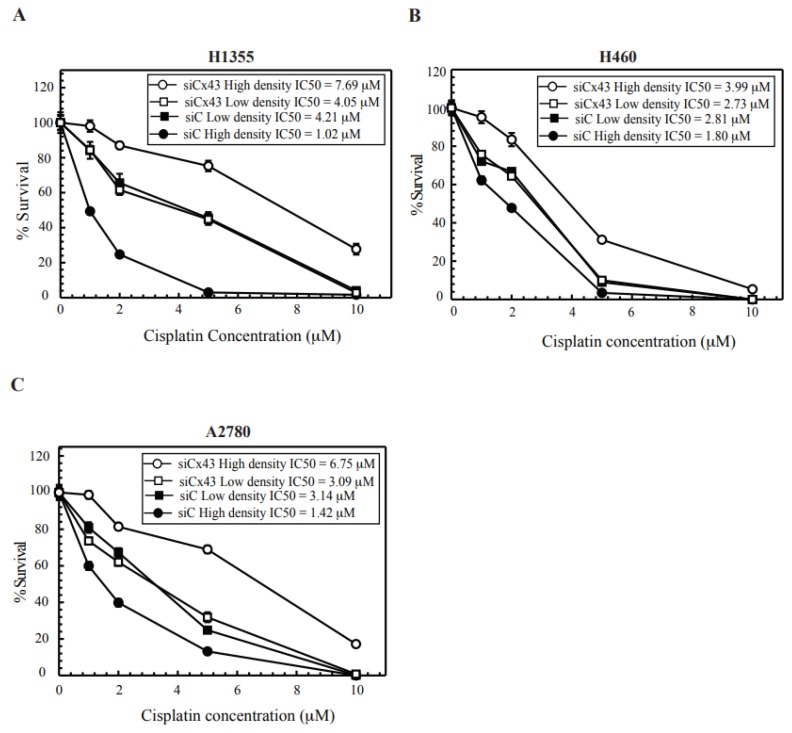
Clonogenic survival at high- and low-density post Cx43 knockdown. (**A**) H1355, (**B**) H460, (**C**) A2780 cells. Non-targeting siRNA transfected (siC) and siCx43 transfected cells were treated to cisplatin at high density and low density and plated for colony survival as described in the methods section. Calculated IC_50_ values are represented in each figure for each cell line. Values are represented as mean ± SEM from three independent experiments, each plated in triplicate.

Increased cisplatin cytotoxicity at high density is consistent with results observed with radiation and recent reports on cisplatin [5,12,18,23,24,25]. Such density-dependent cytotoxicity implicated the role of GJ formation and GJIC. We next tested the role of GJs in this enhanced cytotoxicity and knocked down Cx43 in H1355, H460 and A2780 cells. As seen in Figure 2, H1355 cells exhibited increased expression of Cx43 when compared to H460 cells. In Figure 3A–C, when Cx43-downregulated cells (see Figure S3B,C for knockdown levels) are treated with cisplatin at high density resistance to cisplatin is observed while the colony survival curve for Cx43 knock down at low density resembled the Control siRNA at low density. We observed that knockdown of Cx43 in H1355 and A2780 cells led to decreased dye transfer when compared to control siRNA (Supplemental Figure S3E) demonstrating a disruption in gap junction activity. These results not only contribute to the evidence that GJIC mediates cisplatin cytotoxicity at high density but also that Cx43 expression and functional GJ formation is critical for cisplatin cytotoxicity. In addition, the increased resistance in the siCx43 cells treated at high density compared to siCx43 cells treated at low density, may represent additional density dependent functions of Cx43 in regulating toxicity aside from GJIC, including potential roles in mediating cell signaling events through protein-protein interactions [26]. In Supplemental Figure S2A, we tested the levels of Cx43 in cells plated at low or high density. The levels showed no difference, which suggests that it is their function rather than expression levels that lead to differences in survival.

### 2.4. Cisplatin Treatment Produces DNA DSBs in Bystander Cells

The radiation-induced “bystander effect” (RIBE) has been demonstrated experimentally both in vitro and in vivo. It has been shown that it is manifested via GJIC from irradiated cells to non-irradiated cells [12,24]. RIBE leads to DNA DSB formation in neighboring cells. A recent study showed that cell stresses other than radiation can also lead to the formation of DNA DSBs. UVC damage was also shown to induce DNA DSBs in bystander cells [27]. These reports prompted us to test if cisplatin toxicity in bystander cells is due to the formation of DNA DSBs.

DSBs are a very lethal form of DNA damage and their persistence could lead to genomic instability, cell death and also tumorigenesis. The histone variant γ-H2AX is phosphorylated at serine 139 on exposure to ionizing radiation and forms distinct nuclear foci at sites of DSBs. The disappearance of these foci is correlated with the repair of DSBs. γ-H2AX foci also form on exposure to cisplatin, although cisplatin does not directly induce DSBs. It is known that it is the processing of ICLs that can result in the formation of DSBs [28].

**Figure 4:**
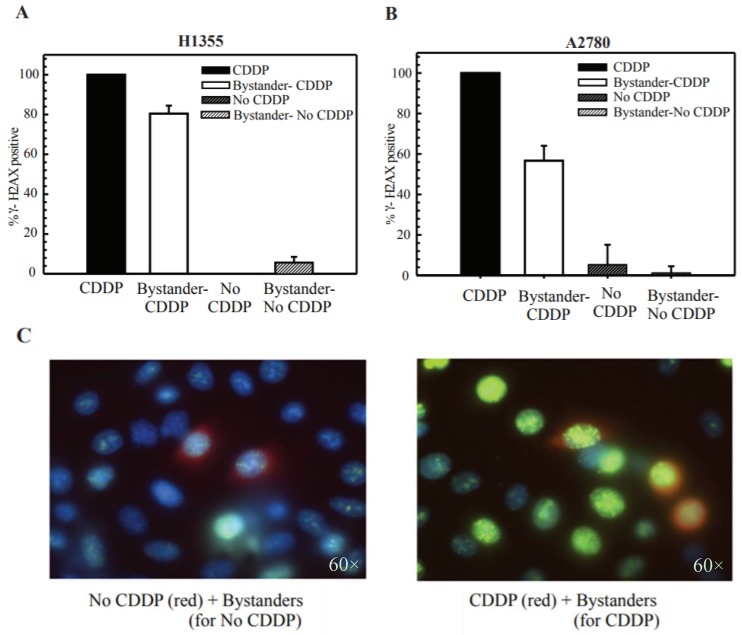

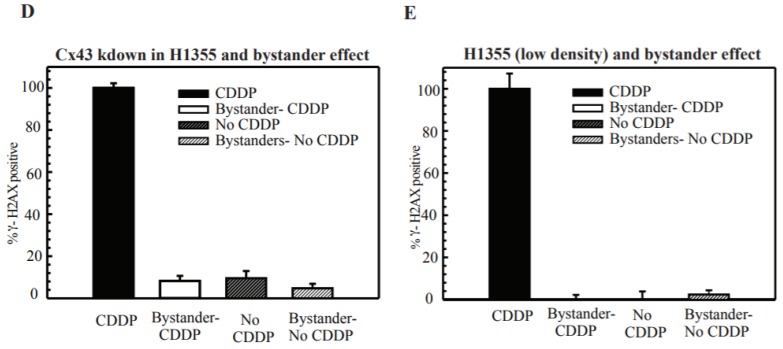
Positive γ-H2AX foci indicating DNA DSB formation in bystander cells post-cisplatin treatment. (**A**) H1355 and (**B**) A2780 cells were divided into 2 populations—labeled with vital cell tracker dye or left unstained. Labeled cells were either treated or left untreated (control) to cisplatin (CDDP) and then mixed with unstained untreated cells and then visualized for positive γ-H2AX foci by immunostaining. Values are represented as percent above background ± S.D. from 3 independent experiments. (**C**) Representative image from H1355 cells. Left panel is without cisplatin/vehicle while right panel is cisplatin treated. Blue—DAPI, red/orange—cell tracker orange, green—γ-H2AX foci merged. (**D**,**E**) DSB formation in H1355, Cx43 knockdown cells (**D**) and cells plated at colony density (**E**). In both cases, cell were divided into 2 populations—labeled with vital cell tracker dye or left unstained. Labeled cells were either treated or left untreated (control) to cisplatin and then mixed with unstained untreated cells and then visualized for positive γ-H2AX foci by immunostaining. Values are represented as percent above background ± S.D. from 3 independent experiments.

In radiation treated cells, different methodologies have been used to study the BE, for example, individual cells are targeted using a single particle microbeam accelerator and in this fashion, biological effects can be recorded in irradiated and unirradiated bystander cells. In another case, cells were irradiated and immediately mixed with unirradiated cells [27]. Since cisplatin cannot be targeted to specific cells like irradiation, we decided to vitally label cells prior to cisplatin treatment. The treated cells were then mixed immediately post treatment with untreated unlabeled cells to study the biological effects. In Figure 4, H1355 (4A) and A2780 (4B) cells were labeled with 5 μM of vital cell tracker dye, cell tracker orange. The cells were divided into two labeled populations: P1C and P1 for each cell line. P1C cells were treated with cisplatin (cis-diamminedichloroplatinum; CDDP) for 2 h and P1 cells were left untreated. All cell groups were trypsinized and mixed and seeded onto coverslips. After 24 h, these cells were fixed and stained for the phosphoepitope of γ-H2AX.

In both H1355 and A2780 cells, when cisplatin treated P1C cells were mixed with untreated P2 cells, increased γ-H2AX foci formation as well as more nuclei with foci above the background levels was observed (background levels—1–5 foci for H1355 and 1–3 foci for A2780, Figure 4C—H1355 representative image). However, when untreated P1 cells were mixed with untreated P2 cells, a regular foci distribution was observed. These data suggest that in the presence of cisplatin treated cells, the bystander cells receive a “toxic” signal or damage which manifests as DNA DSBs. These results parallel results observed with radiation or UVC bystander effect (BE) [27]. 

### 2.5. DSB Production in Bystander Cells Depends on Functional GJIC

Next, we further confirmed the role of GJIC in DSB formation in bystander cells. We either knocked down Cx43 in all three cell populations: P1C, P1 and P2 or mixed these populations at a density where they could not form functional GJs to evaluate the contribution of GJIC. In Figure 4D, Cx43 was knocked down in H1355 cells, and we saw reduced γ-H2AX foci in the bystander cells. We chose H1355 cells as we achieved the best Cx43 knockdown in these cells and they have a high basal level of Cx43 expression. Previous reports have shown that residual levels may also contribute to a substantial level of BE [7]. In Figure 4E, H1355 cisplatin treated (P1C) or untreated (P1) cells were plated at low or colony forming density with the P2 bystander cells. We observed the same results as with Cx43 KD (Figure 4D). These data suggest that GJIC and specifically Cx43 in these cell line models have a significant role in the manifestation of the BE.

Cisplatin has a molecular weight of 300 Da and is small enough to diffuse through GJ channels and potentially spread to the untreated cells. Thus, to rule out the possibility that cisplatin could be the molecule diffusing through GJs, we performed immunofluorescence with the cisplatin-DNA GG-intrastrand specific antibody. If cisplatin is diffusing through GJs, then the unlabeled untreated bystander P2 cells would also form the major cisplatin-DNA intrastrand adducts in addition to the treated P1C cells. In H1355 cells, we treated P1C cells with cisplatin for a period of 2 h to induce adducts or left them untreated (P1 cells) and then trypsinized and mixed both P1C and P1 cells with the untreated P2 cells at a high density. The next day, the cells were fixed and stained with the antibody specific to the GG-intrastrand adducts. In Figure S4, the representative image shows that only cisplatin treated H1355 cells have the damage-specific staining and not the bystander cells, demonstrating that cisplatin does not cross GJs and induce BE in the neighboring cells.

### 2.6. Functional Gap Junction Communication Potentiates Cisplatin Cytotoxicity in DNA Repair Deficient Cells

**Figure 5:**
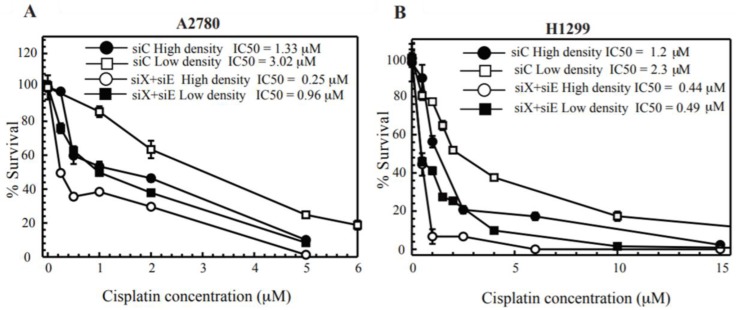
Clonogenic survival in ERCC1/XPF knockdown cells on cisplatin treatment at low and high density of cells. (**A**) A2780 and (**B**) H1299. Non-targeting siRNA (siC) and ERCC1-XPF siRNA transfected cells (siX + siE) transfected cells were treated to cisplatin at high density and low density and plated for colony survival as described in the methods section. Calculated IC_50_ values are represented in each figure for each cell line. Values are represented as mean ± SEM from three independent experiments.

Jensen and Glazer [5] have shown that a proficiency in the NHEJ complex Ku70/80 could lead to cisplatin sensitivity only when cells are treated at gap junction forming density. They also showed that the Ku-initiated death signal required DNA-PK activation and was transmitted from cell to cell through GJs. This study was the first to show that DNA repair status in one cell could affect the survival in another cell. ERCC1-XPF is an important enzyme and has been shown to be important in all aspects of cisplatin-DNA repair and mediate platinum efficacy [29]. Thus, we tested whether positive GJ interactions could potentiate cisplatin toxicity in cisplatin-DNA repair deficient cells using ERCC1-XPF knockdown cells. In previous studies, we have downregulated ERCC1-XPF at ~90–95% levels and shown that this highly sensitizes cisplatin treated cancer cells [29]. In Figure 5A, ERCC1-XPF knockdown in A2780 cells when treated with cisplatin at high cell density leads to enhanced toxicity when compared to ERCC1-XPF knockdown cells at low density. These results suggest that positive GJ formation and communication could increase cisplatin cytotoxicity in cancer cells targeted with DNA repair inhibitors or in DNA repair deficient cells. When compared to the original IC_50_ of A2780 cells at low density, there is a ~10 fold increase in toxicity. H1299 cells (Figure 5B) exhibited a modestly increased toxicity at high density and have reduced levels of Cx43 (when compared to A2780s and H1355s). H1299 is a p53 deficient cell line and differences in p53 expression could also affect cell survival and DNA repair efficiency following ERCC1-XPF knockdown. From these experiments, we observed that there is an increased bystander killing in cells deficient in cisplatin-DNA damage repair. This suggests that the level of DNA damage and DNA damage signaling could help propagate the death signal in the BE.

## 3. Discussion

Our study evaluates the GJ dependent component of cisplatin cytotoxicity and its impact on the bystander cancer cells. Lung and ovarian cancer patients receive a platinum doublet (cisplatin or carboplatin) combined with another chemotherapeutic or radiation therapy. Some of these patients will be non-responders or will eventually develop resistance to the treatment [30,31]. Multiple mechanisms of cisplatin resistance have been explained over the years, of which repair of cisplatin− DNA adducts is considered to be the most important and clinically relevant [29]. The clinical effectiveness of poly (ADP-ribose) polymerase (PARP) inhibitors for BRCA-deficient tumors deficient in homologous recombination has greatly impacted treatment response and overall patient survival [32]. These studies also suggest that understanding mechanisms that may enhance toxicity in DNA repair targeted or in cells that are deficient for key factors involved in repair of cisplatin-induced DNA damage could be clinically beneficial.

Recent reports have suggested that cisplatin cytotoxicity can be enhanced by modulating GJIC [5,18,19,25]. Thus, to better understand the contribution of GJ mediated effects on cisplatin cytotoxicity, we evaluated different cancer cell lines in our study. We observed that cisplatin cytotoxicity increased with an increase in cell density during treatment in both lung and ovarian cancer cells (Figure 1). We next studied the expression of Cx43, which is the most ubiquitously expressed Cx and is detected in most cell types [33]. Cx43 expression was especially elevated in cisplatin sensitive ovarian cancer parental cell lines and reduced in the daughter-derived cisplatin resistant cells (Figure 2). We also show that Cx43 is often hypermutated in human tumors (Figure 2E). It is important to note that not only is expression of Cx43 important, but also the functionality of Cx43 is likely key for eliciting the bystander effect. Mutations in Cx43 are strewn throughout the gene and many mutations are found in regions of functional significance including in the C-terminal domain, critical for proper gap junction assembly and for key protein interactions [34]. Aside from expression of Cxs which are often downregulated or improperly localized in solid tumors, mutations in Cx43 may also limit the permeability of GJs which could also impact the bystander effect [35]. More work is needed to better understand how specific mutations in Cx43 may alter the bystander effect, either via blocking GJIC, decreasing protein stability, disallowing proper hemichannel formation, altering the Cx43 interactome, or changing subcellular localization.

Next, we also showed that the increased density dependent toxicity relied on the formation of functional GJs and not just the expression of Cx43 (Figure 3 and Figure S3A). It has been previously reported that irradiation of cells induced the formation of γ-H2AX foci in bystander cells. Thus, we performed co-culturing studies and observed that mixing untreated cells with cisplatin treated cells induced DNA DSBs in the bystander cells (Figure 4). We further showed that DSBs are not induced when Cx43 is downregulated or cells are at a low density where they cannot form gap junctions (Figure 4). In our studies, DSB formation was not observed in cells at a low density thus requiring further studies to understand the contribution of Cx hemichannels (Figure 4). Future studies are warranted to elucidate the accumulation of DSBs in the bystander cells and assess the damage-foci kinetics to determine if they are resolved.

We also showed that cisplatin (Supplemental Figure S4) is not the molecule that traversed the GJs and induced the DNA damage in bystander cells. The “death” signal has been widely debated and investigated; several molecules have been implicated as the toxic molecule inducing cell death. Glutathione (GSH) is a likely candidate (307 Da) to protect against cisplatin induced damage by detoxification of cisplatin [23]. Another report showed that the death signal passing through GJs in cells treated with cisplatin may be produced by DNA-dependent protein kinase/Ku70/Ku80 signaling [5]. The actual mechanism of how DNA-PK/Ku70/Ku80 mediates this response is still unclear but could involve DNA damage phosphorylation cascades or oxidative stress. Reactive oxygen species (ROS), or other signaling molecules, including ATP, cAMP, IP3 and calcium are also likely candidates. Inflammatory stress signals have also been implicated as candidates for the bystander signal. Thus, further studies with inhibition of molecules may further clarify the contribution from these factors [36]. A recent review suggests a model where targeted cells produce ‘signals’ that traverse through GJs and modulate the redox status in recipient cells. This modulated redox status in conjunction with DNA replication, mimics DNA damage responses and can lead to cell death in recipient cells [37]. Finally, we also show that functional GJIC further enhanced sensitivity in cells deficient in the ERCC1/XPF DNA repair enzyme, required for repair of platinum-induced DNA damage. In Figure 5, we showed that positive GJ formation further sensitized ERCC1/XPF deficient cells to cisplatin. These results warrant further exploration of synergy between the relative competency of tumor cell GJIC and a DNA repair defect that may drive the BE through increasing DNA damage and thus increasing the signal being propagated via GJs. 

Thus, functional GJIC enhanced cisplatin cytotoxicity by propagating the “toxic” signals among coupled cancer cells that never received the chemotherapeutic agent. In a tumor, it would be essential to have functional GJ interactions to yield a greater chemotherapeutic response. Several cancers exhibit mutations or down regulation of Cxs. This suggests that Cx expression could be further developed as a biomarker for assessing chemotherapeutic response or a therapeutic target. It is common knowledge that tumors often acquire resistance to platinum-based chemotherapies. Because tumors are heterogeneous, in the context of Cx43, resistance could arise through a selection of cells that harbor reduced Cx43 expression levels, potentially including cancer stem cells as has been described in various tumor types including gliomas which could contribute to a more aggressive, cisplatin-resistant phenotype [38]. Several studies have suggested that multiple tumor types differentially modulate GJs or GJIC especially during the transition to an invasive or metastatic phenotype [36]. Finally, multiple biological and pharmacological agents have been shown to regulate GJs, such as, suberoylanilide hydroxamic acid (SAHA), carotenoids, green tea components (epicatechin), vitamin D, lycopene (a component of tomatoes) and resveratrol (antioxidant in red wines) [39,40,41,42]. Pharmacological enhancement of GJIC or Cx43 expression would be an ideal target for enhancing cisplatin efficacy in patients by increasing the bystander effect thereby increasing the clinical impact of platinum treatment in patients. In fact there have been preclinical studies assessing the potential of using a GJIC enhancer to supplement cisplatin efficacy in vitro and in vivo, however more work is likely needed before a trial in humans is warranted [8,43]. However, the large number of natural agents that likely regulate GJs would make ideal, likely non-toxic, potential candidates for supplementing current standard of care treatments such as cisplatin to enhance tumor response. Supplementing cisplatin chemotherapy with these agents could further enhance cytotoxicity or targeted overexpression of Cx43 protein in tumor cells may benefit platinum therapy [44]. Therapeutic manipulation would likely be most beneficial in overcoming cisplatin resistance associated with decreases in Cx43 expression, in tumors harboring low basal levels of Cx43 expression, or potentially in tumors harboring certain Cx43 mutations. A better understanding of the basic biology of Cxs in relation to the bystander effect is needed in order to fully implement a proper method to evaluate which patients would most greatly benefit from this type of therapeutic enhancement. However, in terms of Cx43 mutations in tumors, more work must be done to better understand the effects of these mutations on GJIC and Cx43 activity in general in order to understand whether agents that influence gap junctions may be effective in increasing GJIC in tumors harboring Cx43 mutations. Our work highlights and supports previous work on the role of gap junctions and the importance of understanding the mechanisms that maintain and mediate cisplatin sensitivity. Importantly, our data suggest new avenues toward better treatment outcomes and survival in cancer patients.

## 4. Materials and Methods

### 4.1. Colony Survival Assay

Colony formation was assessed by a colony-forming assay adapted from Jensen and Glazer [5] for high and low cell density corresponding to conditions in which gap junction formation is permitted or not, respectively. Cells were left untransfected, transfected with control siRNA or transfected with siRNA against Cx43 or ERCC1-XPF wherever mentioned. For the high-density condition, cells were seeded such that they were between 95 to 100% confluent monolayer at the time of drug exposure. Cells were treated with cisplatin for 2 h, washed with phosphate buffered saline (1× PBS), trypsinized, counted and 300–500 cells were seeded into 60 mm dishes and allowed to form colonies. Fresh medium was added when needed. For the low-density condition, 300–500 cells were seeded onto 60 mm dishes and the next day treated with cisplatin for 2 h, after treatment they were incubated for colony formation. Colonies were fixed with 95% methanol and stained with 0.2% crystal violet. Colonies with ≥50 cells were counted using a light microscope. Cell survival was expressed as the ratio of the average number of colonies in drug treated cells versus control cells ×100. The experiment was performed in triplicate for each drug concentration. We also assessed if the trypsinization process post treatment at high density affected survival. For this we plated 5000–10,000 cells at colony forming density. The next day we treated them with cisplatin and post treatment, trypsinized, counted and seeded 300–500 cells in 60 mm dishes for colony formation. The results obtained from this survival assay corresponded to the low-density survival results (Supplemental Figure S2). Experiments were conducted such that both low density and high density cells were treated with cisplatin at approximately the same time after plating and for the same duration.

### 4.2. Chemicals

Cisplatin [*cis*-diammine-dichloroplatinum (II)] was purchased from Sigma–Aldrich (St. Louis, MO, USA). The antibodies were monoclonal α-tubulin (T5168, Sigma, St Louis, MO, USA), Connexin43 antibody from Invitrogen (Waltham, MA, USA), monoclonal anti-phospho γ-H2AX (clone JBW301, Millipore, Burlington, MA, USA), Alexa 488-conjugated goat anti-mouse and anti-rat (Molecular Probes, Eugene, OR, USA). For StaRT-PCR, primers that amplify Cx43/GJA1 and β-actin (control) were obtained from Gene Express (Toledo, OH, USA). β-Actin forward primer 5′-CCCAGATCATGTTTGAGACC-3′; reverse primer 5′-CCATCTCTTGCTCGAAG TCC-3′. Connexin43/GJA1 forward primer 5′-AGCAGTCTTTTGGAG TGACCAGCAACTTTG-3′; reverse primer 5′-CATGCAATGAAGCTGAACATGACCGTAGTT-3′.

### 4.3. Cell Culture

NSCLC cell lines, H1299 (provided by Dr. Randall Ruch, University of Toledo), H1355, H460 (provided by Dr. James C. Willey, University of Toledo) and ovarian cancer cell lines, 2008, 2008/C13 (provided by Dr. Stephen Howell, UCSD), A2780, A2780/C30 (provided by Thomas Hamilton, Fox Chase Cancer Center) were maintained in RPM1 1640 supplemented with 10% FBS in the presence of penicillin (100 IU/mL) and streptomycin (100 μg/mL). Cells were grown at 37 °C in a 5% CO_2_ incubator.

### 4.4. siRNA Sequence and Transfections

siRNA smart pools designed to target human ERCC1, XPF and Cx43 (catalogue numbers L-006311-00, L-019946-00 and L-011042-00, respectively) were purchased from Dharmacon RNA Technologies (Lafayette, CO, USA). A non-targeting siRNA pool was used in control experiments (catalogue number D-001810-10-20). Cells were seeded in six-well plates (density 2.5 × 10^5^ cells/well) in antibiotic free media. Two transfections were done at 24 h interval in each cell line to knockdown Cx43 or ERCC1-XPF according to the manufacturer’s protocol. 

### 4.5. Western Blot

At indicated time points post-transfection, the cells were centrifuged, washed with PBS, and lysed on ice for 30 min in lysis buffer (10 mM Tris (Thermo Fisher Scientific; Waltham, MA, USA), pH 8.0, 120 mM NaCl (Thermo Fisher Scientific), 0.5% NP-40 (US Biological; Salem, MA, USA), 1 mM EDTA (Thermo Fisher Scientific) with protease inhibitors (0.5 M phenyl methyl sulfonyl fluoride (PMSF), 1 mg/mL leupeptin, 1mg/mL pepstatin) (Sigma; St. Louis, MO, USA). Equal amounts of protein were loaded and electrophoresed on 10% SDS–polyacrylamide gel. The proteins were transferred onto PVDF membrane (Immobilon transfer membrane, Millipore; Burlington, MA, USA). After electroblotting, the membranes were blocked with Tris-buffered saline with Tween 20 (1 M Tris–HCl (Thermo Fisher Scientific), pH 7.5, 150 mM NaCl, and 0.5% Tween 20 (Thermo Fisher Scientific) containing 2% non-fat dry milk. Primary antibodies recognizing Cx43 or α-tubulin were diluted in blocking buffer and incubated for 30 min. The membranes were then washed, incubated with the appropriate secondary antibodies in a blocking buffer for 30 min, and washed again. The blotted proteins were detected using enhanced chemiluminescence detection system (0.1 M Tris, pH 8.5 (Thermo Fisher Scientific), 12.5 mM luminol (Sigma), 0.2 mM p-coumaric acid (Sigma), 10 μL 30% hydrogen peroxide (Thermo Fisher Scientific).

### 4.6. RNA Isolation, Reverse Transcription and Transcript Abundance

Cells were lysed with 1 mL of TRIzol reagent and the total RNA was extracted following the manufacturer’s protocol. The total RNA extracted from each cell line was reverse transcribed with oligo dT primer and M-MLV-RT as described previously [44,45]. Transcript levels were quantified using the previously described StaRT-PCR protocol. Briefly, a mixture of internal standard competitive template (SYSTEM 1, Gene Express, Inc., Wilmington, NC, USA) was included in a master mix with cDNA and PCR reagents (dNTPs etc.). The use of internal standards allows comparing data from different experiments giving a highly reproducible, standardized, quantitative measurement of transcript levels. In these studies, β-actin (ACTB) was used as a loading control gene. The master mix was aliquoted into tubes containing each gene-specific primer (ACTB and Cx43). PCR was carried out in a Rapidcycler (Idaho Technology Inc., Salt Lake City, UT, USA) with each reaction mixture subjected to 35 cycles each of 5 s denaturation at 94 °C, 10 s of annealing at 58 °C and 15 s of elongation at 72 °C. PCR products were separated and quantified electrophoretically by the Agilent 2100 Bioanalyzer (Agilent Technologies Inc., Santa Clara, CA, USA) using the DNA 1000 Assay kit. Following electrophoresis, a ratio of the endogenous PCR products (or native template, NT) to the internal standard (competitive template) was taken to calculate molecules of NT in the reaction. Each transcript abundance value was normalized to ACTB and values are reported as target gene mRNA/10^6^ ACTB mRNA. All experiments were performed in triplicate.

### 4.7. Lucifer Yellow Dye-Transfer Assay

The Lucifer yellow (Invitrogen, Waltham, MA, USA) dye transfer assay was performed as previously described [15]. Briefly, cells at complete monolayer were treated with 0.05% Lucifer yellow in 2 mL of DMEM + 10% FBS, linear scrapes were made through the monolayer using a scalpel blade. Treated cells were incubated at 37 °C for 5 min to allow dye uptake, and then dye containing media was removed and cells were washed 2–3 times thoroughly with PBS, fixed with formalin and resuspended in PBS. Dye transfer from cells at the edge of the scrape to the neighboring cells (as a measure of gap junction communication) was visualized using an Eclipse T2000-U microscope (Nikon, Melville, NY, USA) at 20× (summarized in Figure S3D).

### 4.8. Gamma-H2AX Phosphorylation for DNA DSB Measurement

H1355 and A2780 (Cx43 knockdown, control siRNA or untransfected wherever mentioned) cells were trypsinized and divided into three populations per condition per cell line: (1) P1C: Labeled with vital cell tracker dye and cisplatin treated; (2) P1: Labeled with vital cell tracker dye; (3) P2: Untreated and no label. The cells were labeled by a vital cell tracker dye called cell tracker orange according to the manufacturer’s protocol (Invitrogen). The P1C cells were treated with cisplatin for 2 h at 50% survival determined concentrations for all cell lines tested, washed, trypsinized and mixed with trypsinized P2 cells plated onto coverslips. Similarly, the P1 cells were left untreated and mixed with P2 cells plated onto coverslips. The cells were mixed such that P1C or P1 cells were in a ratio of 1:9 with P2 cells and these cells were 85 to 100% confluent for the assay. The next day, the cells were washed with Hank’s balanced salt solution, fixed with freshly prepared 3.7% methanol-free paraformaldehyde for 15 min on ice and permeabilized with 0.3% Triton-X-100 in PBS and blocked with 10% goat serum in PBS. For detecting phosphorylated form of γ-H2AX, cells were incubated for 1h with the monoclonal anti γ-H2AX (1:1000, Millipore) followed by incubation with Alexa-488 goat anti-mouse antibody (1:1000, Molecular Probes) diluted in 10% goat serum in PBS. Cells were washed and counterstained with DAPI for 5 min.

Coverslips were mounted with DAKO mounting medium onto slides and the edges were sealed with nail polish. Images were visualized using a Nikon Eclipse T2000-U microscope at 60× or 100× (when required) oil immersion objective. Foci were counted in 100 randomly chosen cells per condition per cell line per experiment and results are expressed as % γ-H2AX foci per nuclei. Error bars indicate standard deviation and the data were collected from three individual experiments.

### 4.9. Immunofluorescence with Cisplatin-DNA Intra-Adduct Specific Antibody

Immunofluorescence with cisplatin-DNA intrastrand adduct specific antibody was performed as follows: H1355 cells were trypsinized and divided into three populations per cell line as described above for γ-H2AX immunofluorescence—P1C, P1 and P2. The labeled cisplatin treated P1C or labeled untreated P1 cells were mixed with P2 cells respectively and plated onto 60 mm dishes on coverslips for 80 to 100% confluence. The next day, cells were washed with PBS and fixed with freshly prepared 3.7% methanol-free paraformaldehyde for 15 min on ice and permeabilized with 0.3% Triton-X-100 in PBS and the DNA was denatured using 2N HCl for 20 min at room temperature. The denatured cells were blocked for 1-2h at room temperature with 20% fetal bovine serum (FBS) in washing buffer (0.1% Triton X-100 in PBS). For detecting cisplatin-DNA intrastrand adducts we used the ICR4 (provided by Dr. Mike Tilby) antibody diluted in 1% BSA (1:500) and incubated for 1 h at room temperature followed by incubation with the secondary (1:1000, Sigma) diluted in 1% BSA for 2 h at room temperature. Coverslips were mounted with DAKO mounting medium onto the slides. Images were captured by Confocal Microscopy at the Advanced Microscopy and Imaging center at University of Toledo with assistance from Dr. Andrea Kalinoski (Figure S4).

### 4.10. Mutation Frequency and Survival Analysis

The frequency of somatic mutations in genes of interest as well as information on hypermutated and non-hypermutated tumors was extracted from TCGA studies (http://cancergenome.nih.gov/) using cBioPortal [46]. Survival analysis was performed using kmplotter.org. GJA1 affymetrix probe 201667_at was used and tumor expression was stratified by high vs. low expression using the “auto select best cutoff” function [47].

## 5. Conclusions

The data presented in this paper show that cisplatin efficacy appears to vary depending upon Cx43 expression when cells are treated in high density cell cultures. Furthermore, cisplatin efficacy is increased when cells can form gap junctions (high Cx43). While cisplatin treatment does not alter transfer of dye between cells, cisplatin itself is not the death signal that traverses gap junctions. Additionally, we observed that decreasing Cx43 expression through genetic knockdown approaches decreased cisplatin efficacy only in high density cell cultures. Upon cisplatin treatment, bystander cells accumulate the presence of γ-H2AX, a marker indicative of DNA DSBs. Finally, knockdown of the DNA endonuclease ERCC1-XPF further enhances cisplatin sensitivity at both GJ-forming and non GJ-forming densities, suggesting a potential synergistic effect between GJIC and DNA repair capacity in terms of cisplatin treatment.

## Supplementary Materials

The following are available online at http://www.mdpi.com/2072-6694/10/10/368/s1, Figure S1: Schematic representing how clonogenic assays were performed using high and low density cell cultures. Figure S2: Cisplatin treatment at low density followed by splitting and replating of cells for colony survival, Figure S3: Cx43 expression, knockdown time course and dye transfer test, Figure S4: Immunofluorescence with cisplatin-intrastrand adduct specific antibody.

## Abbreviations

NSCLC	Non-small cell lung cancer
GJ	Gap junction
GJIC	Gap junction intercellular communication
ICL	Interstrand crosslinks
DSBs	Double strand breaks
Cx43	Connexin-43
ERCC1	Excision repair cross-complementation group 1
XPF	Xeroderma pigmentation group F
siRNA	Small interfering RNA
StaRT–PCR	Standardized reverse transcription–polymerase chain reaction
ACTB	β-Actin
RIBE	Radiation induced bystander effect
BE	Bystander effect
SAHA	Suberoylanilide hydroxamic acid

## References

[1] Cooley M.E., Davis L., Abrahm J. (1994). Cisplatin: A clinical review. Part II—Nursing assessment and management of side effects of cisplatin. Cancer Nurs..

[2] Cooley M.E., Davis L.E., DeStefano M., Abrahm J. (1994). Cisplatin: A clinical review. Part I—Current uses of cisplatin and administration guidelines. Cancer Nurs..

[3] Cohen S.M., Lippard S.J. (2001). Cisplatin: From DNA damage to cancer chemotherapy. Prog. Nucleic Acid Res. Mol. Biol..

[4] Wernyj R.P., Morin P.J. (2004). Molecular mechanisms of platinum resistance: Still searching for the Achilles’ heel. Drug Resist. Updat..

[5] Jensen R., Glazer P.M. (2004). Cell-interdependent cisplatin killing by Ku/DNA-dependent protein kinase signaling transduced through gap junctions. Proc. Natl. Acad Sci. USA.

[6] Wu D., Li B., Liu H., Yuan M., Yu M., Tao L., Dong S., Tong X. (2018). In vitro inhibited effect of gap junction composed of Cx43 in the invasion and metastasis of testicular cancer resistanced to cisplatin. Biomed. Pharmacother..

[7] Su M., Zhang Q. (2017). Deficiency of gap junction composed of connexin43 contributes to oxaliplatin resistance in colon cancer cells. Oncol. Lett..

[8] Ding Y., Nguyen T.A. (2012). Gap Junction Enhancer Potentiates Cytotoxicity of Cisplatin in Breast Cancer Cells. J. Cancer Sci. Ther..

[9] Garcia-Rodriguez L., Perez-Torras S., Carrio M., Cascante A., Garcia-Ribas I., Mazo A., Fillat C. (2011). Connexin-26 is a key factor mediating gemcitabine bystander effect. Mol. Cancer Ther..

[10] Di X., Bright A.T., Bellott R., Gaskins E., Robert J., Holt S., Gewirtz D., Elmore L. (2008). A chemotherapy-associated senescence bystander effect in breast cancer cells. Cancer Biol. Ther..

[11] Alexandre J., Hu Y., Lu W., Pelicano H., Huang P. (2007). Novel action of paclitaxel against cancer cells: Bystander effect mediated by reactive oxygen species. Cancer Res..

[12] Blyth B.J., Sykes P.J. (2011). Radiation-induced bystander effects: What are they, and how relevant are they to human radiation exposures?. Radiat. Res..

[13] Yakovlev V.A. (2015). Role of nitric oxide in the radiation-induced bystander effect. Redox Biol..

[14] Azzam E.I., De Toledo S.M., Little J.B. (2003). Oxidative metabolism, gap junctions and the ionizing radiation-induced bystander effect. Oncogene.

[15] Azzam E.I., De Toledo S.M., Little J.B. (2001). Direct evidence for the participation of gap junction-mediated intercellular communication in the transmission of damage signals from alpha-particle irradiated to nonirradiated cells. Proc. Natl. Acad Sci. USA.

[16] Vinken M., Vanhaecke T., Papeleu P., Snykers S., Henkens T., Rogiers V. (2006). Connexins and their channels in cell growth and cell death. Cell. Signal..

[17] Mesnil M., Crespin S., Avanzo J.L., Zaidan-Dagli M.L. (2005). Defective gap junctional intercellular communication in the carcinogenic process. Biochim. Biophys. Acta.

[18] Kalvelyte A., Imbrasaite A., Bukauskiene A., Verselis V.K., Bukauskas F.F. (2003). Connexins and apoptotic transformation. Biochem. Pharmacol..

[19] Peterson-Roth E., Brdlik C.M., Glazer P.M. (2009). Src-Induced Cisplatin Resistance Mediated by Cell-to-Cell Communication. Cancer Res..

[20] Trosko J.E., Ruch R.J. (2002). Gap junctions as targets for cancer chemoprevention and chemotherapy. Curr. Drug Targets.

[21] Jamieson S., Going J.J., D’Arcy R., George W.D. (1998). Expression of gap junction proteins connexin 26 and connexin 43 in normal human breast and in breast tumours. J. Pathol..

[22] El-Fouly M.H., Trosko J.E., Chang C.C. (1987). Scrape-loading and dye transfer. A rapid and simple technique to study gap junctional intercellular communication. Exp. Cell. Res..

[23] Hong X.T., Wang Q., Yang Y., Zheng S.P., Tong X.H., Zhang S.Z., Tao L., Harris A.L. (2012). Gap junctions propagate opposite effects in normal and tumor testicular cells in response to cisplatin. Cancer Lett..

[24] Hei T.K., Zhou H., Ivanov V.N., Hong M., Lieberman H.B., Brenner D.J., Amundson S.A., Geard C.R. (2008). Mechanism of radiation-induced bystander effects: A unifying model. J. Pharm. Pharmacol..

[25] He B., Tong X.H., Wang L.Z., Wang Q., Ye H., Liu B., Hong X.T., Tao L., Harris A.L. (2009). Tramadol and Flurbiprofen Depress the Cytotoxicity of Cisplatin via Their Effects on Gap Junctions. Clin. Cancer Res..

[26] Sorgen P.L., Trease A.J., Spagnol G., Delmar M., Nielsen M.S. (2018). Protein(-)Protein Interactions with Connexin 43: Regulation and Function. Int. J. Mol. Sci..

[27] Sokolov M.V., Smilenov L.B., Hall E.J., Panyutin I.G., Bonner W.M., Sedelnikova O.A. (2005). Ionizing radiation induces DNA double-strand breaks in bystander primary human fibroblasts. Oncogene.

[28] Olive P.L., Banath J.P., Durand R.E. (1990). Heterogeneity in radiation-induced DNA damage and repair in tumor and normal cells measured using the "comet" assay. Radiat Res..

[29] Arora S., Kothandapani A., Tillison K., Kalman-Maltese V., Patrick S.M. (2010). Downregulation of XPF-ERCC1 enhances cisplatin efficacy in cancer cells. DNA Repair (Amst).

[30] Simon G.R., Ismail-Khan R., Bepler G. (2007). Nuclear excision repair-based personalized therapy for non-small cell lung cancer: From hypothesis to reality. Int. J. Biochem. Cell. Biol..

[31] Simon G.R., Begum M., Bepler G. (2008). Setting the stage for tailored chemotherapy in the management of non-small cell lung cancer. Future Oncol..

[32] Brown J.S., Kaye S.B., Yap T.A. (2016). PARP inhibitors: The race is on. Br. J. Cancer.

[33] Chevallier D., Carette D., Segretain D., Gilleron J., Pointis G. (2013). Connexin 43 a check-point component of cell proliferation implicated in a wide range of human testis diseases. Cell. Mol. Life Sci..

[34] Leithe E., Mesnil M., Aasen T. (2018). The connexin 43 C-terminus: A. tail of many tales. Biochim. Biophys. Acta Biomembr..

[35] Aasen T., Mesnil M., Naus C.C., Lampe P.D., Laird D.W. (2016). Gap junctions and cancer: Communicating for 50 years. Nat. Rev. Cancer.

[36] Yamasaki H., Krutovskikh V., Mesnil M., Tanaka T., Zaidan-Dagli M.L., Omori Y. (1999). Role of connexin (gap junction) genes in cell growth control and carcinogenesis. CR Acad Sci. III.

[37] Klammer H., Mladenov E., Li F., Iliakis G. (2015). Bystander effects as manifestation of intercellular communication of DNA damage and of the cellular oxidative status. Cancer Lett..

[38] Yu S.C., Xiao H.L., Jiang X.F., Wang Q.L., Li Y., Yang X.J., Ping Y.F., Duan J.J., Jiang J.Y., Ye X.Z. (2012). Connexin 43 reverses malignant phenotypes of glioma stem cells by modulating E.-cadherin. Stem Cells.

[39] Stahl W. (2016). Carrots, tomatoes and cocoa: Research on dietary antioxidants in Dusseldorf. Arch. Biochem. Biophys..

[40] Trosko J.E. (2005). The role of stem cells and gap junctions as targets for cancer chemoprevention and chemotherapy. Biomed. Pharmacother..

[41] Kelsey L., Katoch P., Ray A., Mitra S., Chakraborty S., Lin M.F., Mehta P.P. (2014). Vitamin D3 regulates the formation and degradation of gap junctions in androgen-responsive human prostate cancer cells. PLoS ONE.

[42] Ogawa T., Hayashi T., Tokunou M., Nakachi K., Trosko J.E., Chang C.C., Yorioka N. (2005). Suberoylanilide hydroxamic acid enhances gap junctional intercellular communication via acetylation of histone containing connexin 43 gene locus. Cancer Res..

[43] Shishido S.N., Nguyen T.A. (2012). Gap junction enhancer increases efficacy of cisplatin to attenuate mammary tumor growth. PLoS ONE.

[44] Weaver D.A., Crawford E.L., Warner K.A., Elkhairi F., Khuder S.A., Willey J.C. (2005). ABCC5, ERCC2, XPA and XRCC1 transcript abundance levels correlate with cisplatin chemoresistance in non-small cell lung cancer cell lines. Mol. Cancer.

[45] Willey J.C., Crawford E.L., Jackson C.M., Weaver D.A., Hoban J.C., Khuder S.A., DeMuth J.P. (1998). Expression measurement of many genes simultaneously by quantitative RT-PCR using standardized mixtures of competitive templates. Am. J. Respir. Cell. Mol. Biol..

[46] Cerami E., Gao J., Dogrusoz U., Gross B.E., Sumer S.O., Aksoy B.A., Jacobsen A., Byrne C.J., Heuer M.L., Larsson E. (2012). The cBio cancer genomics portal: An open platform for exploring multidimensional cancer genomics data. Cancer Discov..

[47] Gyorffy B., Surowiak P., Budczies J., Lanczky A. (2013). Online survival analysis software to assess the prognostic value of biomarkers using transcriptomic data in non-small-cell lung cancer. PLoS ONE.

