# Supplementary material for: Gap Junction Intercellular Communication Positively Regulates Cisplatin Toxicity by Inducing DNA Damage through Bystander Signaling
Source: Cancers (Basel). 2018 Oct 2;10(10):368. doi: 10.3390/cancers10100368 (PMC6210410; doi:10.3390/cancers10100368)
Supplement: Supplementary file 1 [file cancers-10-00368-s001.pdf]

SUPPLEMENTAL DATA

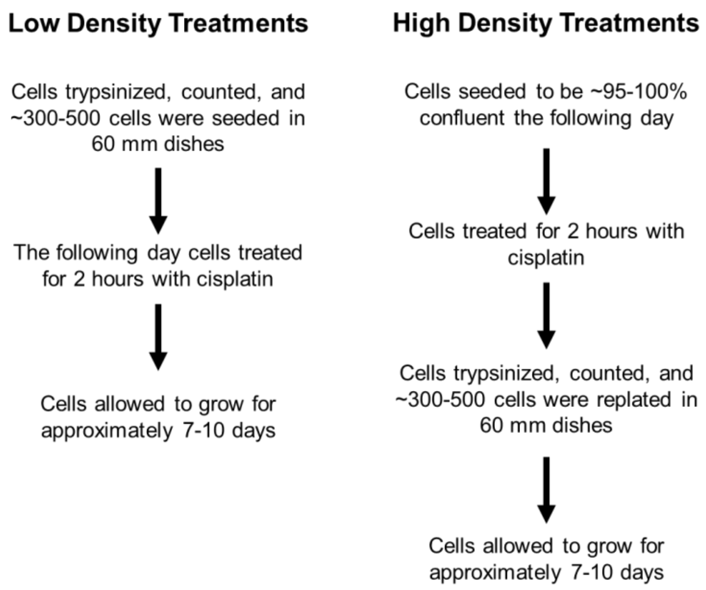

Figure S1. Schematic representing how colony formation assays were performed for low and high density cisplatin treatments. Schematic represents the number of cells seeded, the relative time frame for treatment, and the length of time colonies were allowed to grow.

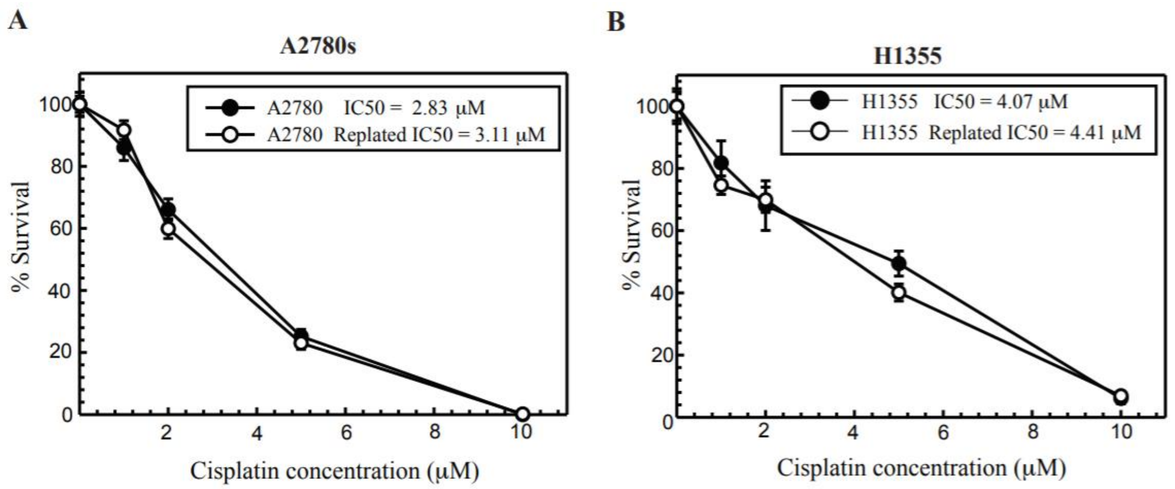

Figure S2. Cisplatin treatment at low density followed by splitting and replating of cells for colony survival - A) A2780 and B) H1355 cells. Clonogenic survival was performed at low density cisplatin treatment and then cells were split, counted and replated to 60 mm dishes for colony formation. Calculated IC50 values are represented in each figure for each cell line. Values are represented as mean ± SEM from independent experiments.

A

Cx43 - High Density (HD) or Low Density (LD)

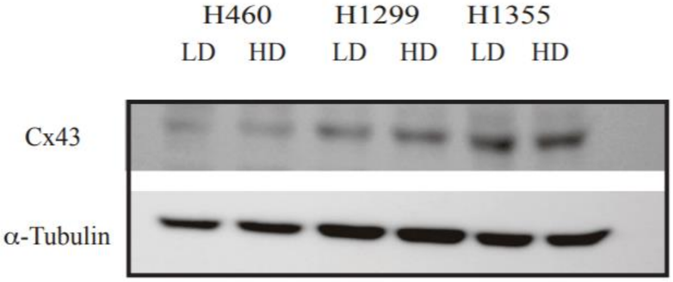

B

Cx 43 knockdown -H1355

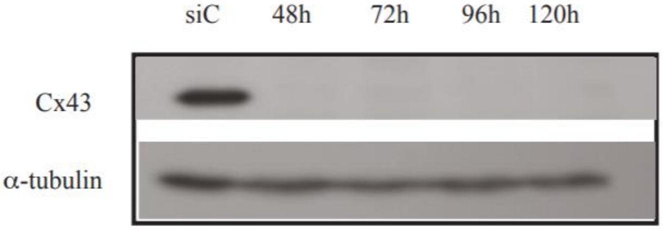

C

Cx43 knockdown -A2780

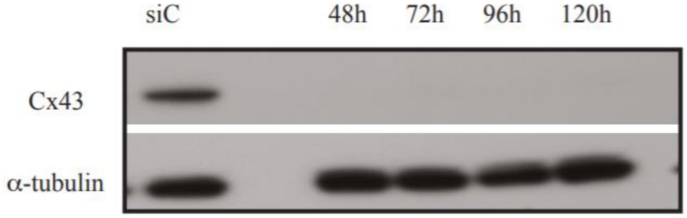

D

Lucifer yellow dye transfer

Cell Line	Positive transfer	Effect on transfer post cisplatin treatment
H1299	Yes - transfers to 2-4 cells	No effect
H1355	Yes	No effect
H460	Yes - transfers to 2-3 cells	N/A
A2780	Yes	N/A

H1355 Untreated

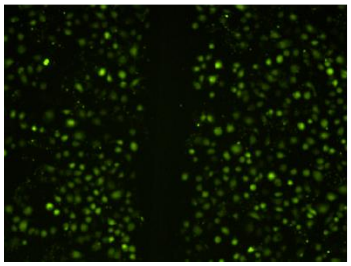

H1355 + Cisplatin

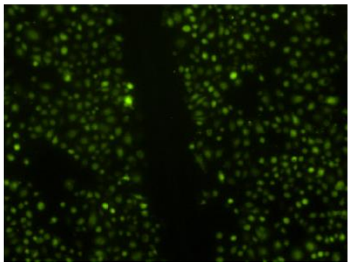

E

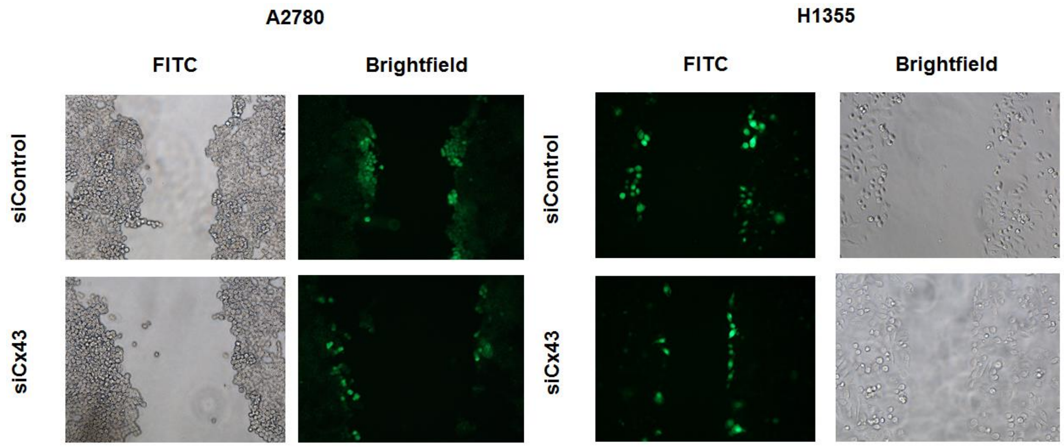

Figure S3. Cx43 expression, knockdown time course and dye transfer test. A) Cx43 protein expression at high- and low-density cell plating for NSCLC cells – H460, H1299 and H1355. B) H1355 and C) A2780 cells Cx43 knockdown. Whole cell lysates were prepared at each timepoint post transfection for knockdown and control cells. Non-targeting siRNA denoted as siC, and Cx43 knockdown timepoints from 48 to 120h post transfection as described in the methods section. Lysates were probed with Cx43 antibody with α-tubulin as a loading control. D) Summary of Lucifer yellow dye transfer assay for NSCLC and A2780 ovarian cancer cells with or without cisplatin treatment and sample images of dye transfer in H1355 cells ± cisplatin treatment. E) Dye transfer with control and Cx43 knockdown in H1355 and A2780 cells.

Immunostaining with cisplatin intrastrand adduct - H1355

A **B**

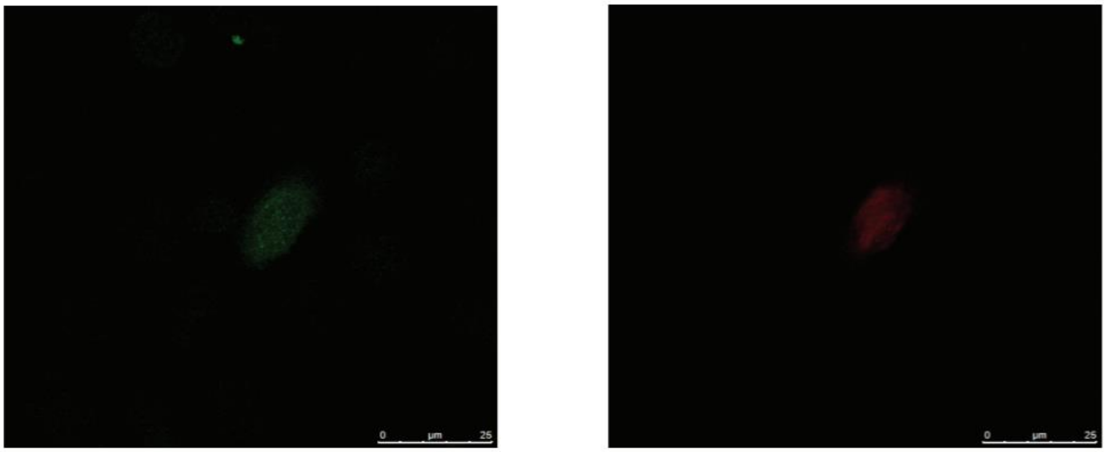

C D

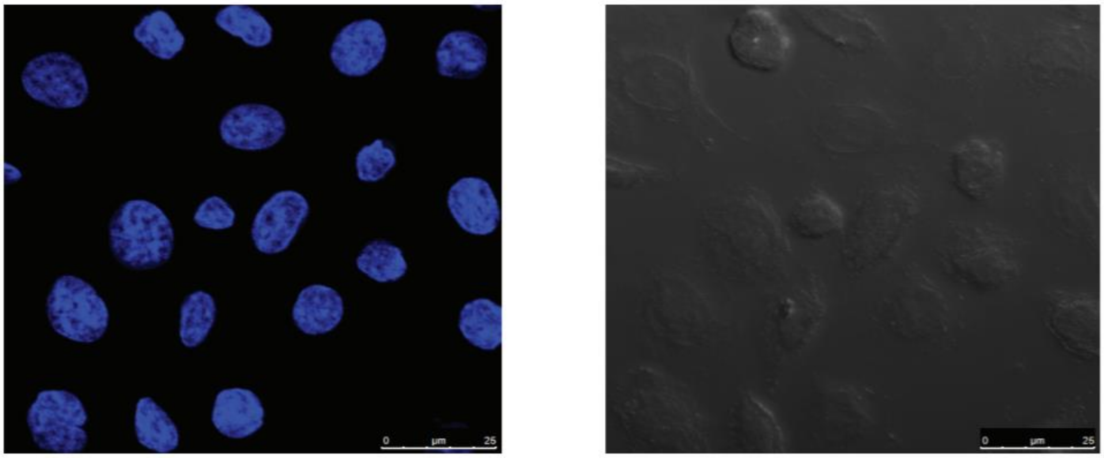

Figure S4. Immunofluorescence with cisplatin-intrastrand adduct specific antibody. In H1355 cells, A) green is the cisplatin-intrastrand adduct specific antibody staining the cisplatin treated cell, B) red labels the cell tracker orange labeled cisplatin treated cells, C) DAPI staining and D) Brightfield.
